# Supplementary material for: A novel ILP framework to identify compensatory pathways in genetic interaction networks with GIDEON
Source: Bioinformatics. 2026 Aug 21;42(Suppl 2):btag385. doi: 10.1093/bioinformatics/btag385 (PMC13501320; doi:10.1093/bioinformatics/btag385)
Supplement: Supplementary file 1 [file btag385_supplementary_data.zip › btag385_Supplementary_Data.pdf]

Supplementary Material for: A Novel ILP Framework to Identify Compensatory Pathways in Genetic Interaction Networks with GIDEON

Jocelyn J. Garcia Kevin M. Yu Catherine Freudenreich Lenore J. Cowen

Department of Computer Science
Tufts University
Medford, MA 02155

1 Supplementary Methods

1.1 Constructing the Nonessential x Nonessential Gene Network

For some pairs of genes a and b, the nonessential double knockout data from [7] contains reciprocal double mutant fitnesses: one corresponding to the cross where a is the query strain and b is the array strain, and another where the roles are reversed. After removing interactions with missing values in either the single or double mutant fitnesses, we use the most lenient suggestion from [6] as follows:

1. Remove both reciprocal interactions when the interactions from [7] have opposite signs (say, a as the query strain has a positive interaction and b as the query strain has a negative interaction).

2. For any remaining duplicate edges, remove the interaction with the higher p-value according to [7].

After removing edges with missing values and following their suggested filtering, the resulting genetic interaction network has 4,459 genes with 6,821,060 edges.

1.2 Adjusting for Experimental Error in the DI-Weighting Scheme

As discussed in [7], a small number of single mutant strains were found to carry a second suppressor mutation, leading to the behavior in the marginal distribution demonstrated by *PHB1* in Figure 2. All strains marked with a suppressor mutation demonstrated different single mutant fitnesses based on whether they were in the array or query strain in the double knockout. Across the data, we identified 37 genes whose single mutant fitnesses differ between the array and query strains, and these 37 genes include all strains with suppressor mutations as well as additional genes whose strains may have carried suppressor mutations as well based on their marginal distributions. The genes with differing single mutant fitnesses, as well as the magnitude of that difference, are as follows: *LTE1* (0.05), *UPS1* (0.22), *NUP170* (0.26), *HCR1* (0.30), *ARV1* (0.31), *RSM25* (0.57), *YIL014C-A* (0.02), *NCE101* (0.01), *PEX32* (0.07), *CBF1* (0.33), *MSC1* (0.31), *THP3* (0.31). *RPL43A* (0.34), *SNF1* (0.27), *VPS53* (0.29), *REI1* (0.18), *RRP6* (0.35), *SLX8* (0.08), *MNN10* (0.26), *PHB1* (0.35), *PAC10* (0.34), *LPD1* (0.12), *DDR2* (0.01), *GTR2* (0.13), *SAE3* (0.02), *ACL4* (0.01), *RMR1* (0.14), *BUB1* (0.24), *ROM2* (0.37), *LHS1* (0.12), *BUD27* (0.13), *SWA2* (0.28), *ARC18* (0.30), *SEC22* (0.31), *MRM2* (0.31), *DCK1* (0.01), *MAC1* (0.02).

When computing the weights and the residual standard deviation s_{res} for these 37 genes in the Distribution-Informed Weighting Scheme, we constructed two separate marginals, each with their own model and s_{res}, based on whether the gene was in the query or array strain in a double knockout. This allowed the model and s_{res} to more accurately reflect the gene's typical behavior in a double knockout.

In [7], there are also a small number of genes (17) with two different array single mutant fitnesses, but these unique values differed by less than 0.025 and therefore do not present a strong enough argument to build separate marginal distributions within the array marginal distribution. That said, in the event of significant discrepancies, one could imagine generalizing the Distribution-Informed waiting scheme to build separate marginal distributions based on the array single mutant fitness, forming more homogenous groups with a more accurate measure of expected gene behavior in that environment.

We last note that the computation of residual standard deviation has a divisor of $N - 2$, so the edge-case where a model is built on less than 3 data points can cause a negative or undefined s_{res}. Though this event did not occur in the data used here (and should not occur with high-throughput SGA data), when deciding which edges to filter, the implementation of GIDEON currently opts to keep any interaction based on less than 3 data points rather than attempt to compute residual standard deviation, with the argument that such few data points do not give a true sense of expected gene behavior.

1.3 Additional Weighting Schemes for Comparison

In addition to comparing GIDEON, LocalCut, and Liany-ILP using our DI weights and the stringent filtering of [7] weighting schemes, we also compared each method performance to the intermediate filtering of [7] as well as the weighting schemes compared by [30] from [20]. As discussed in Section 5 of the main text, LocalCut had superior performance on smaller networks and the use of filtered networks allows for manageable model sizes in GIDEON and Liany-ILP. Costanzo et. al. provided suggestions for filterings of the network described as stringent and intermediate, each offering different balances of false negatives and false positives [7].

- *Original-Stringent*: Retain all edges where $P < 0.05$ and $|\varepsilon| > 0.08$
- *Original-Intermediate*: Retain all edges where $P < 0.05$ and $\varepsilon > 0.16$ or $\varepsilon < -0.12$.

With our DI Weighting Scheme containing 156,428 edges, these two filterings provide an elegant comparison to networks with slightly fewer and slightly more edges, with the Original-Stringent and Original-Intermediate filterings producing networks with 97,162 and 250,081 interactions respectively. We will refer to these weighting schemes as OG-Str and OG-Int.

Beyond the filterings of the original weights, we compared LocalCut performance using the following weighting schemes discussed in [20, 30], where $D_{a,b}$ represents the double mutant fitness of genes a, b and S_a, S_b represent the single mutant fitnesses of genes a, b respectively:

- *Minimum*: $w_{a,b} = D_{a,b} - \min(S_a, S_b)$
- *Logarithmic*: $w_{a,b} = \log_2(D_{a,b}) - (\log_2(S_a) + \log_2(S_b))$
- *Multiplicative*: $w_{a,b} = D_{a,b} - (S_a \cdot S_b)$

Note that we will refer to the weighting schemes as Min-All, Log-All, and Mult-All to represent that the full network of interactions is being used, while the abbreviation "Mult" specifically refers to the multiplicative weighting scheme filtered by edge magnitude to the same size as the DI weights (156,428) . We similarly

refer to the DI weights on the full interaction network without the filtering by residual standard deviation as DI-All. Though [7] stated that they computed their interaction scores using the multiplicative weighting scheme, their data presents a small number of discrepancies. Of the 6,821,060 unique interactions in our network, 20 had weights of 0.0 rather than their expected weights under the multiplicative weighting scheme.

A very small number of interactions (119) also had negative double mutant fitnesses, which we removed from the data only when computing the Logarithmic weighting scheme because a negative double mutant fitness produces an undefined interaction score.

1.4 Illustration of the Impact of Gene-Centered Constraints

We further demonstrate the utility of constraining g_c, the gene used to build the BPM, as the primary contributor to the interaction weight objective function. Consider the BPM in Figure S1a, which is centered on *ATP12* and highlights compensation within the mitochondria. Within this BPM, *ATP12* has 11 mild to moderate negative interactions, demonstrating its role as the primary contributor to the raw ILP output with 25 genes in each module. By constraining *ATP12* as the main contributor, GIDEON pushes the search toward BPMs containing many strong interactions of *ATP12*, exemplified in this case by the inclusion of other mitochondrial genes in the BPM. The left module is enriched for the mitochondrial outer envelope and the right for the ERMES complex.

Upon removal of the constraints forcing *ATP12* as the primary contributor, we find the BPM in Figure S1b, which clearly does not take *ATP12* into consideration when constructing the BPM and superficially appends *ATP12* onto a peak in the response surface of general stress response genes discussed in Section 2.3 of the supplement. This superficial addition takes the form of *ATP12* having *no interactions* with any other gene in the BPM and it thus plays no role in its construction. When a BPM is constructed for every gene, we find that the vast majority follow this superficial addition, necessitating the primary contributor constraints. That said, the elegance of every BPM having *some* primary contributor ensures that this strong BPM can still be found by GIDEON using a more relevant gene.

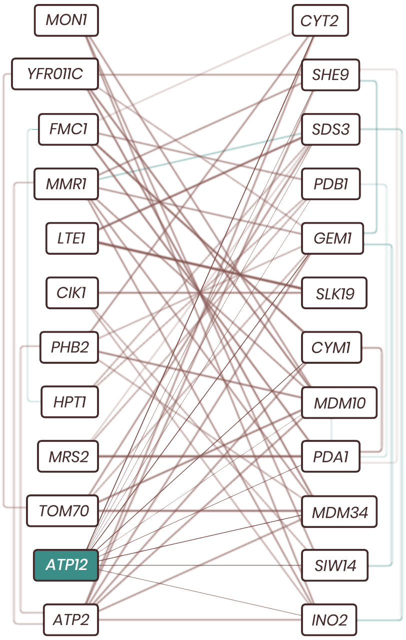

(a) GIDEON BPM after trimming and pruning

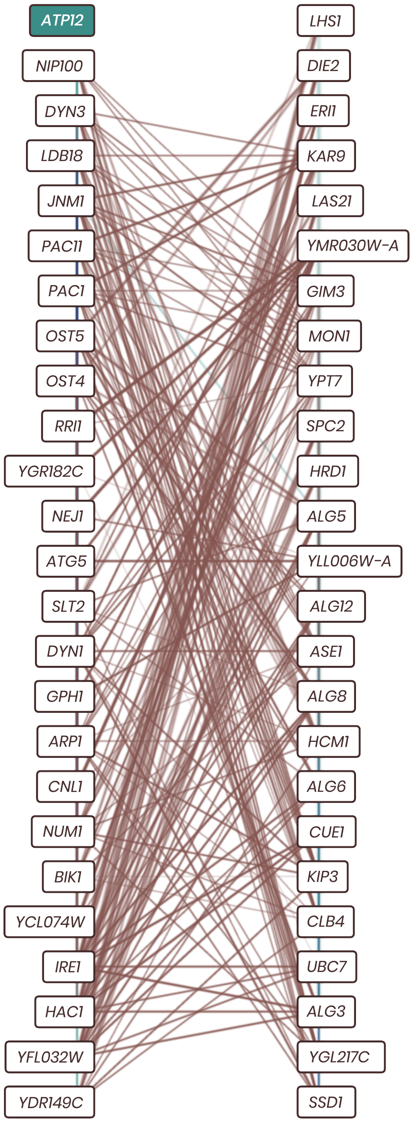

(b) Raw ILP without primary contributor constraints

Figure S1: We present an illustrative example of why the primary contributor constraint is useful. The best weight BPM identified by GIDEON centered on *ATP12*, after trimming and pruning, consists of primarily mitochondrial genes. However, the BPM on the right contains *ATP12* and has much higher weight: this BPM is constructed by taking the highest global weight BPM, and then just appending *ATP12* to one side, where it has no epistasis with any of the other genes in either module. Without the primary contributor constraints on the ILP, it would return the BPM on the right again and again, just adding in each new gene in turn.

2 Supplementary Results

2.1 Ergosterol & Aromatic Amino Acid Biosynthesis BPM Biological Exploration

As hinted in the main text, the BPM shown by Figure 5 suggests ties between ergosterol and aromatic amino acid biosynthesis. Multiple genes in the right module are either directly involved in ergosterol biosynthesis or demonstrate ties to amino acid transport. *ERG3* and *ERG6* are in the ergosterol biosynthesis pathway, with *ERG6* specifically suggested as an antifungal target [13]. *VBA5* and *BAP2* are each plasma membrane proteins involved in amino acid uptake, with *STP1* being the transcription factor of *BAP2* [4].

The left module boasts three shikimate pathway enzymes (*TRP3*, *ARO7*, *TYR1*), a key piece of aromatic amino acid synthesis that has been described as an attractive source of antifungal drug targets [16]. Though YGR259C is a dubious open reading frame, it almost completely overlaps with *TNA1* and thus its interaction profile likely resembles *TNA1* [27]. The role of *TNA1* in related processes further suggests this resemblance, with *TNA1* and *BNA1* each involved in NAD^+ biosynthesis from the aromatic amino acid tryptophan [18]. *TKL1* is also required for aromatic amino acid biosynthesis [26], while *ARV1* relates to ergosterol through involvement in sterol transport and exogenous sterol uptake [13].

Inclusion of amino acid uptake genes with *ERG3* and *ERG6* across from amino acid biosynthesis suggest potential compensatory relationships between the two processes. Previous work has suggested inhibitors of aromatic amino acid biosynthesis as antifungal drugs [16] with tryptophan biosynthesis noted as particularly promising [12]. Though the Jaccard of this BPM is low with all LocalCut and Liany-ILP BPMs, various portions of this BPM were recovered by different LocalCut outputs. The LocalCut BPM with the largest overlap included the genes *ERG6*, *TKL1*, *BAP2*, *STP1*, *ARO7*, *TRP3*, and *TYR1* and was thus most notably missing *ERG3*, *BNA1*, YGR259C, *VBA5* and *LAS21*, instead including more amino acid transport genes such as *TAT1*, *TAT2*, *ARO1*, and *ARO2*. A few small ($<$ 15 genes) LocalCut BPMs contain both *ERG3* and *ERG6* and tended focus on the tryptophan biosynthesis genes *TRP1*, *TRP2*, *TRP3*, *TRP4* but are consistently missing the plasma membrane proteins involved in amino acid uptake that may provide a tangible link between the two processes.* The Liany-ILP BPM with the largest overlap includes *ERG6*, *ARO7*, *TYR1*, *TKL1*, *BAP2*, and *STP2*, but is again missing *ERG3* with minimal additional insight in its 44 additional genes beyond also identifying *ARO1*. Only two Liany-ILP BPMs contain both *ERG3* and *ERG6*, but each BPM contains primarily noise from other processes. Of the 50 genes in each BPM, about three genes show strong ties to aromatic amino acid synthesis and two genes with weaker ties, again missing the tangible link of amino acid uptake genes. Given the evidence provided by the GIDEON BPM in Figure 5 and its pieces recovered by LocalCut, the relationships identified above could thus suggest potential insight into future antifungal drugs.

*We do make the caveat that LocalCut is a randomized algorithm, and BPM collections can vary between runs, though across the LocalCut BPM collection, metrics of number of BPMs and enrichment tend to be quite stable between runs [30]. Here we are comparing the GIDEON BPM collection to the LocalCut BPM collection with the same random seed as produced the results in the main paper.

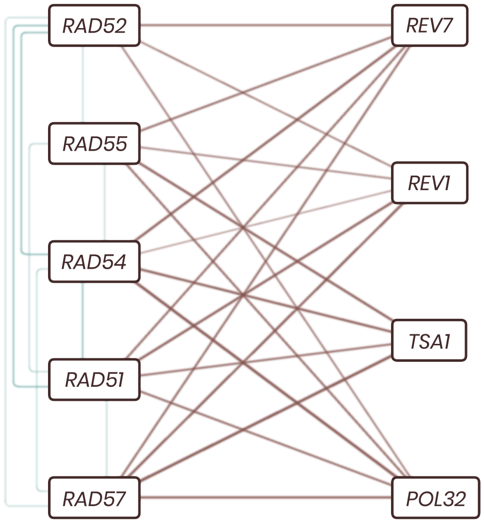

Figure S2: GIDEON identifies a BPM that highlights core pathway genes in the well-established compensation of homologous recombination and translesion synthesis.

2.2 GIDEON Finds Both New and Known Examples of Compensatory Pathways

The number of BPMs found by GIDEON imply that it finds novel BPMs not uncovered by other methods. However, we also show that GIDEON finds some of the best-known BPMs whose modules contain pathways whose compensatory behavior is well known. We show that GIDEON recovers the majority of the strongest LocalCut BPMs, with 93 of the 100 strongest LocalCut BPMs by interaction weight matched by a similar GIDEON BPM (Jaccard > 0.2). As an example of a GIDEON BPM that matches one of the LocalCut BPMs highlighted in [30], and concerning a very well studied redundant pathway in yeast, we highlight the GIDEON BPM with two different DNA damage tolerance pathways that mediate bypass of DNA lesions [9] in Figure S2.

2.3 Frequently Included Genes

We looked at the genes that were most often included across GIDEON BPMs. As has also been noted in previous work [17], many BPMs include housekeeping genes involved in stress response. These genes are not universally present across all BPMs, but intriguingly suggest specific general stress response mechanisms might be involved in successfully switching to backup pathways when a primary pathway is broken. Further biological validation would be needed to confirm this hypothesis. These genes also show up in BPMs for LocalCut and LianyILP, so it is not an artifact of the GIDEON method of constructing BPMs.

We were additionally curious what the functional enrichment of our BPMs would like like when the genes in Table S1 are filtered out of the BPMs (and the 210 BPMs that then have less than 3 genes in a module are removed). We find that the enrichment statistics of the pared down modules are comparable, with only a slight decrease from 92.6% of all GIDEON BPM modules enriched to 91.7%; this is likely caused by the complete removal of strong BPMs primarily comprised of these general stress response genes.

Gene	BPM Count	Relevant Function
IRE1	634	Regulation of unfolded protein response [8]
HAC1	628	Regulation of unfolded protein response [11]
YMR030W-A	618	Protein of unknown function [15]
YFL032W	473	Dubious ORF, overlaps HAC1 [10]
COG8	438	Intra-Golgi vesicle-mediated transport [29]
COG5	396	Intra-Golgi vesicle-mediated transport [29]
YCL060C	367	Merged ORF into MRC1 [5]
COG6	358	Intra-Golgi vesicle-mediated transport [29]
MRC1	344	S-phase checkpoint protein [14]
GLO3	325	GTPase involved in ER-Golgi transport [23]
SGS1	297	Maintains genome integrity [28]
HPT1	277	Purine-containing compound salvage [2]
YDL162C	277	Dubious ORF that overlaps ENT1 [10]
ALG3	272	Glycoprotein biosynthetic process [1]
ALG8	268	N-linked glycosylation [25]
TOP3	267	Regulates mitotic recombination [3]
MEC3	264	DNA damage checkpoint protein [22]
IME1	258	Regulation of transcription by RNA polymerase II [19]
LEO1	252	Transcription elongation by RNA polymerase II [24]
MMS4	249	Recombination and DNA repair [21]

Table S1: The most common genes across GIDEON BPMs, sorted by their frequency. Without implicit limitation on the number of BPMs that can include a given gene or interaction, GIDEON allows for the ubiquity of genes involved in general stress response. The most common genes in GIDEON BPMs with our DI weights tend to specifically play roles in unfolded protein response, Golgi transport, and DNA damage response.

3 Supplementary Figures

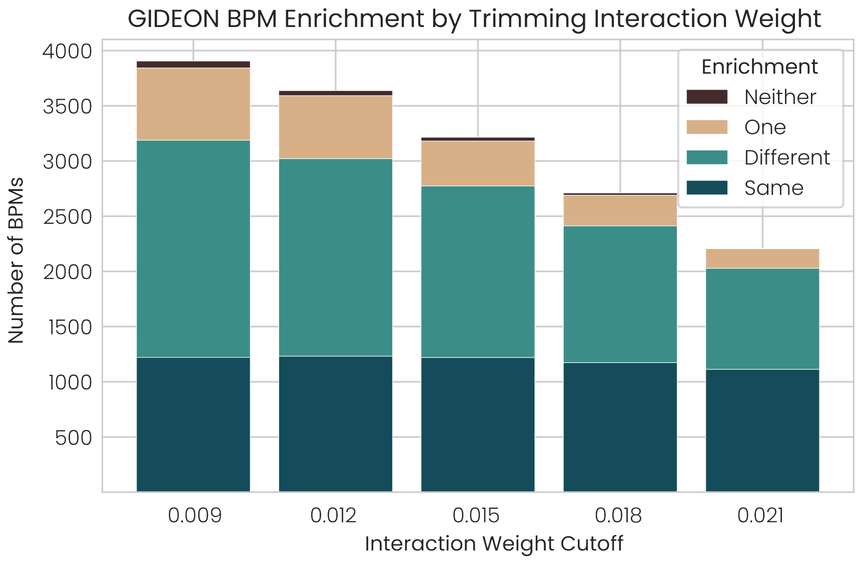

Figure S3: When varying the interaction weight cutoff for the trimming procedure of GIDEON with our DI weights, we identified that a cutoff of 0.015 produced cohesive BPMs itself, with surrounding cutoffs producing cohesive BPMs as well.

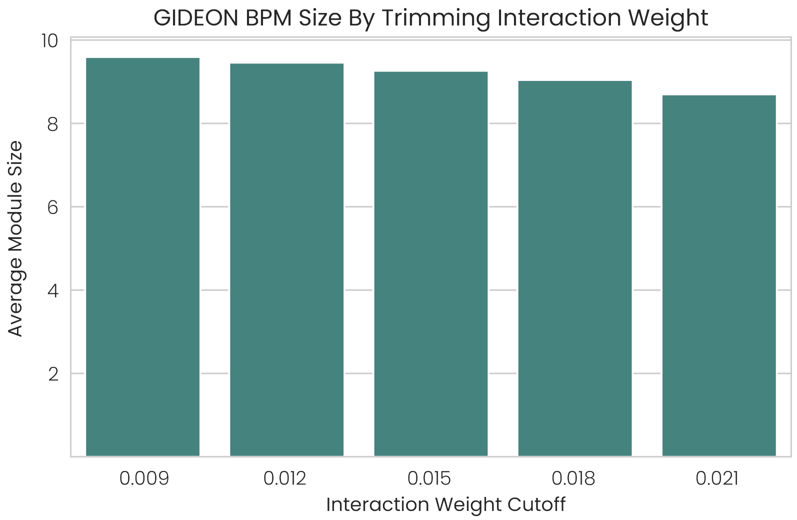

Figure S4: In addition to robust enrichment with a cutoff of 0.015 in Figure S3, we find that varying the interaction weight cutoff has little impact on the size of outputted BPM modules, with the average number of genes only slightly dropping as the cutoff becomes stricter.

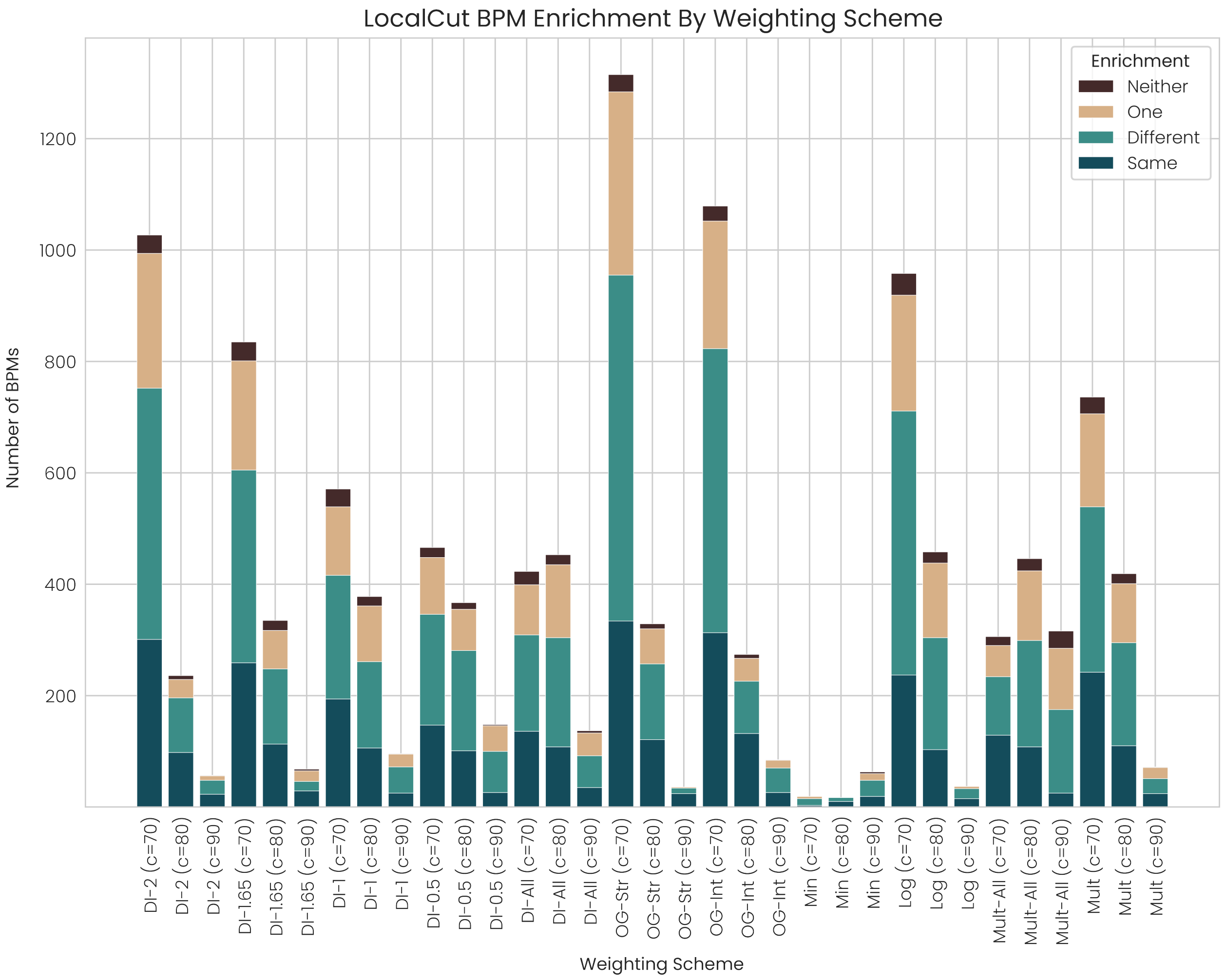

Figure S5: A comparison of LocalCut BPM output quantity and enrichment with different weighting schemes, with three settings of LocalCut's c parameter. OG-Str and OG-Int weights for LocalCut outperform DI weights for LocalCut, but we again note that these methods require additional experimental data and are not generalizable to genetic interaction data. Note that LocalCut with DI weights already outperforms all approaches from [20] compared in [30]. We also compare LocalCut performance with our DI weights at varying s_{res} cutoffs, demonstrating that GIDEON's default value of $s_{res} > 2$ produces also the most BPMs with competitive enrichment for LocalCut. Thus we set s_{res} identically for GIDEON and LocalCut when computing our DI weights for the results in the main paper.

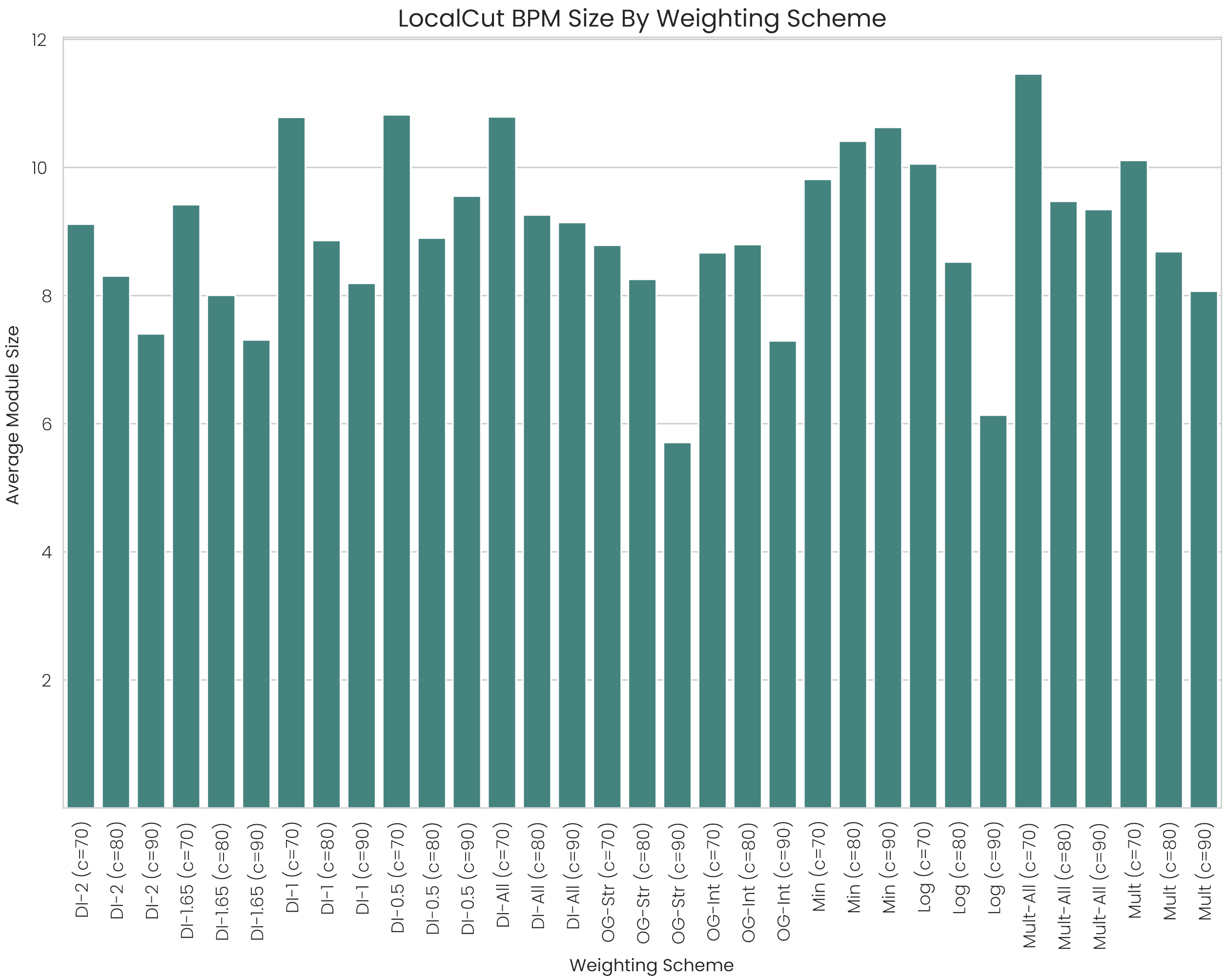

Figure S6: When considering LocalCut BPM output, we tend to favor weighting schemes and LocalCut parameters that produce slightly larger BPMs because they tend to yield more interesting biological insights. We note that our DI weighting scheme had an average module size of approximately 9 genes, the largest of the weighting schemes that produced a notable number of BPMs besides Log-All.

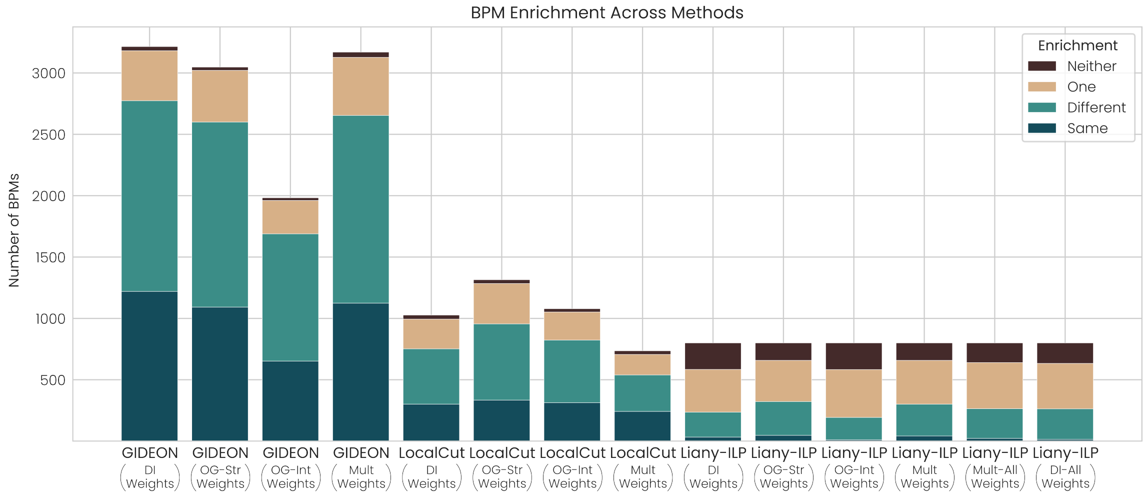

Figure S7: GIDEON substantially outperformed LocalCut and Liany-ILP both within and across weighting schemes. With GIDEON, our DI weighting scheme produced the most BPMs and the strongest enrichment scores, with OG-Str and Mult producing competitive BPM counts and enrichment as well. With LocalCut, the OG-Str weights identified 288 more BPMs than the DI weights, but we reiterate that additional experimental data is needed to build the OG-Str and OG-Int networks. With Liany-ILP, the OG-Str and Mult weights identified slightly more BPMs enriched for the same and different terms, but the poor enrichment scores of Liany-ILP make us hesitant to view these results as reflective of weighting scheme quality. Given that our DI weighting scheme outperformed OG-Sig with GIDEON and the results on LocalCut and Liany-ILP are competitive, our new approach is robust across methods, suggesting its potential for robust results across applications of epistasis data as well.

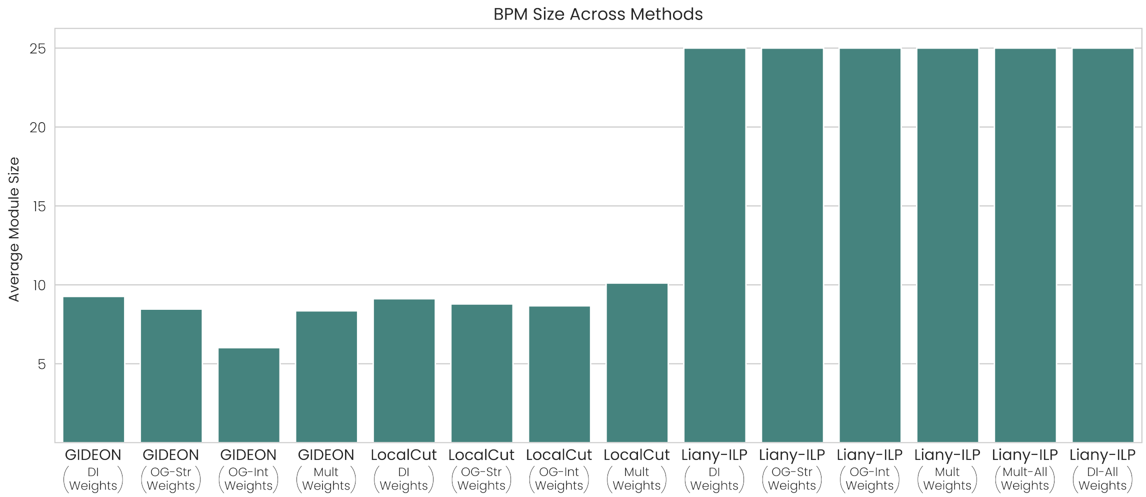

Figure S8: While GIDEON and LocalCut maintained average BPM sizes of roughly nine genes in each module, Liany-ILP struggled with almost always producing BPMs with the maximum number of genes in each module. Initially exhibited by GIDEON and motivating the trimming procedure, the decision in Liany-ILP to ignore edges within modules exacerbated the problem of many genes in the network at least minimally improving the objective function, leading to the large BPMs.

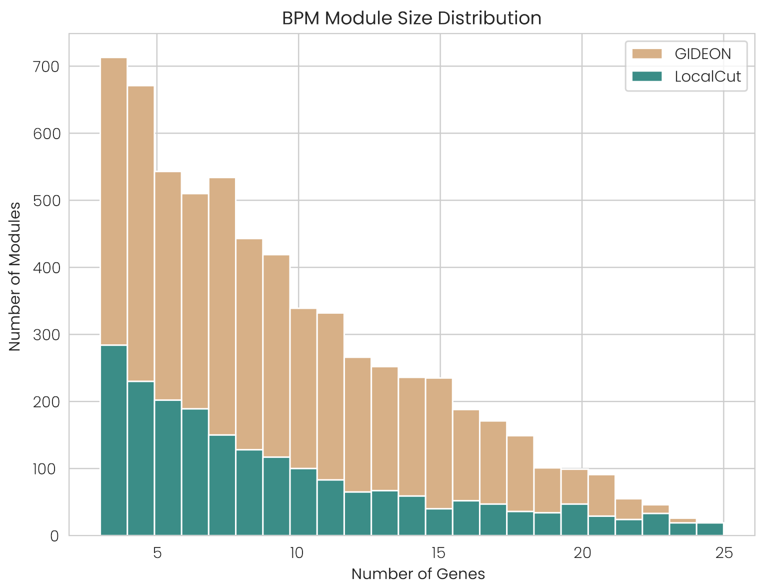

Figure S9: With our DI weights, GIDEON returns BPMs with the same average module size as LocalCut, demonstrating that two stages of pruning produce BPMs of reasonable size from the original ILP outputs with 25 genes in each module. Liany-ILP is not included because nearly every module had 25 genes.

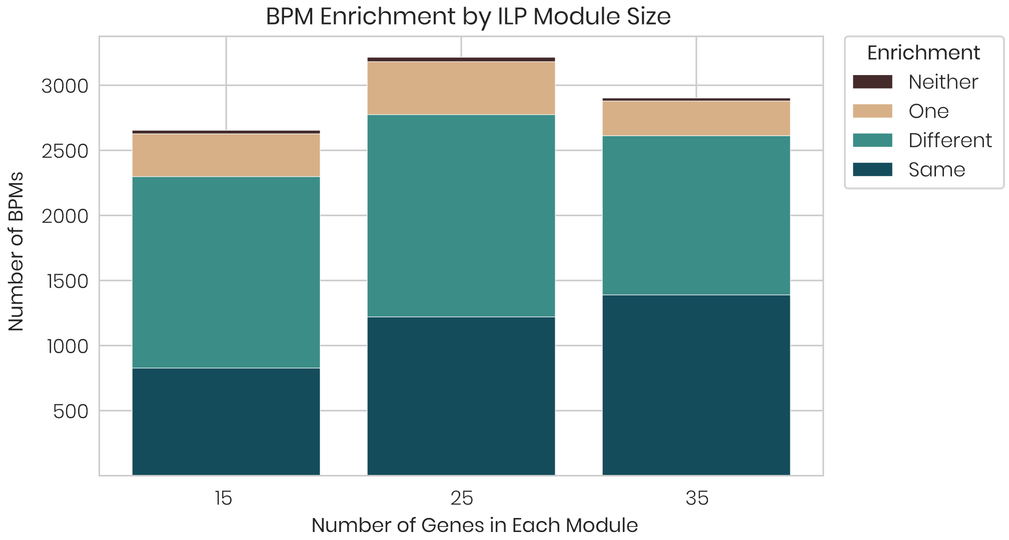

Figure S10: We performed an ablation varying the number of genes in each pathway that the ILP is initially asked to return. We set this to 25 to match LocalCut, but here we see what happens to the collection of the BPMs when this is set to 15 or to 35. We find that setting this to 25 produces the largest collection of BPMs, justifying our choice for choosing 25 beyond just wanting our results to be comparable to LocalCut. When going to 35 from 25 genes, the resulting BPM collection returns 548 modules with $>$ 25 genes, making these results not comparable to LocalCut, but we note that increasing the number of genes the ILP is initially allowed to return may be worth exploring further: the average number of genes in the final GIDEON BPM collection increases from 9.2 to 12 after final trim, so it will find slightly larger BPMs.

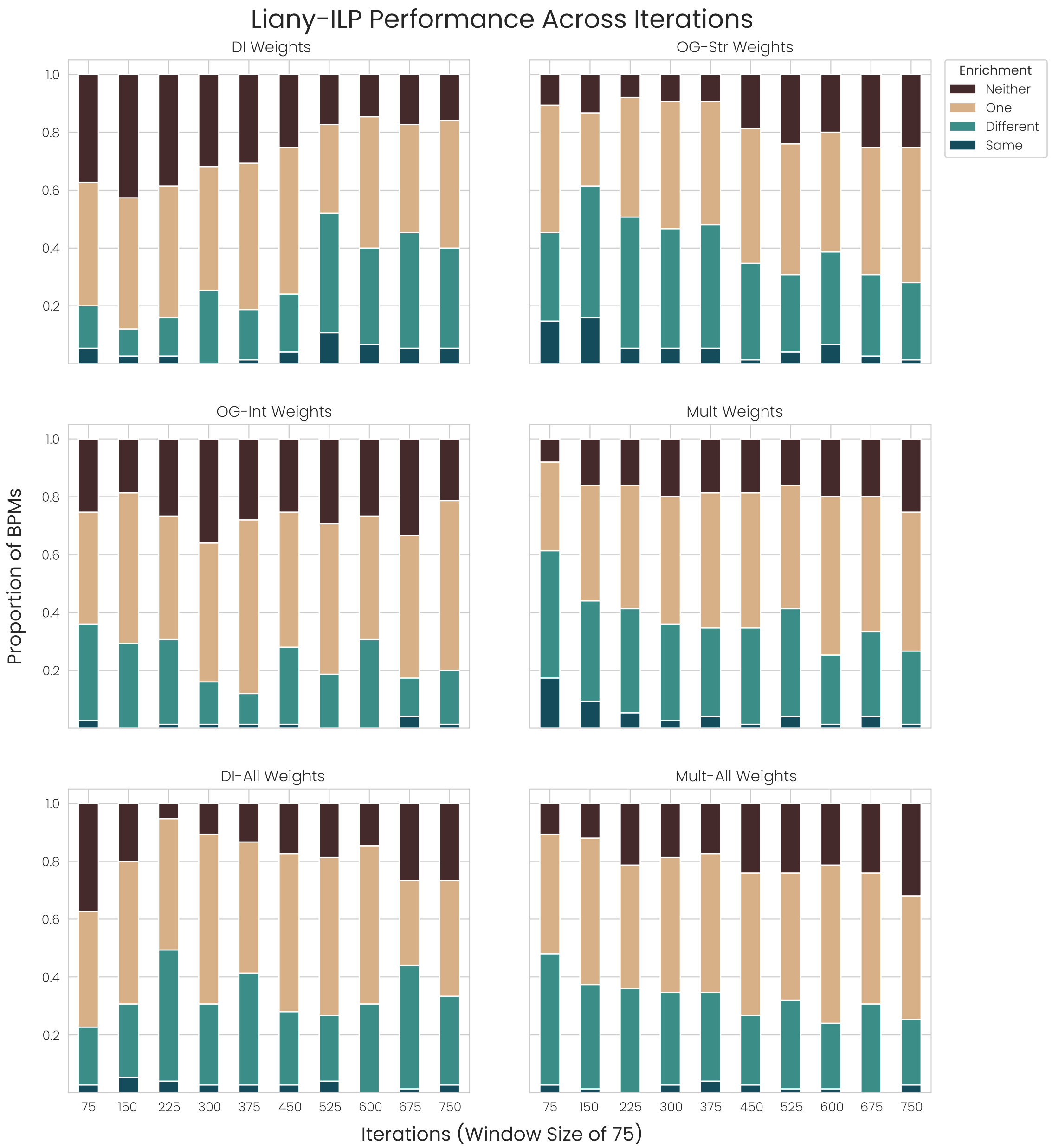

Figure S11: Given the sequential and iterative nature of Liany-ILP, the BPMs identified become worse as the algorithm continues. In other words, the first BPMs will tend to be best and BPM quality will fall as K (the number of BPMs) increases because Liany-ILP removes all edges after finding a BPM. We observe above that the BPM quality of Liany-ILP is poor from the first 75 BPMs and tends to fall from then on, demonstrating that higher values of K are unlikely to improve output. Because the edge removal is an inherent limit on the number of BPMs found, we also run Liany-ILP on Mult-All and DI-All but do not observe improvement in BPM quality. We also not that running Liany-ILP with the DI weights until the graph was empty produced 618 additional BPMs with only 18 enriched for the same term, further demonstrating that a larger K is almost certainly not beneficial. The sequential nature of Liany-ILP also lends itself to tedious runtimes, with identifying 833 BPMs using the Mult weights requiring over two days.

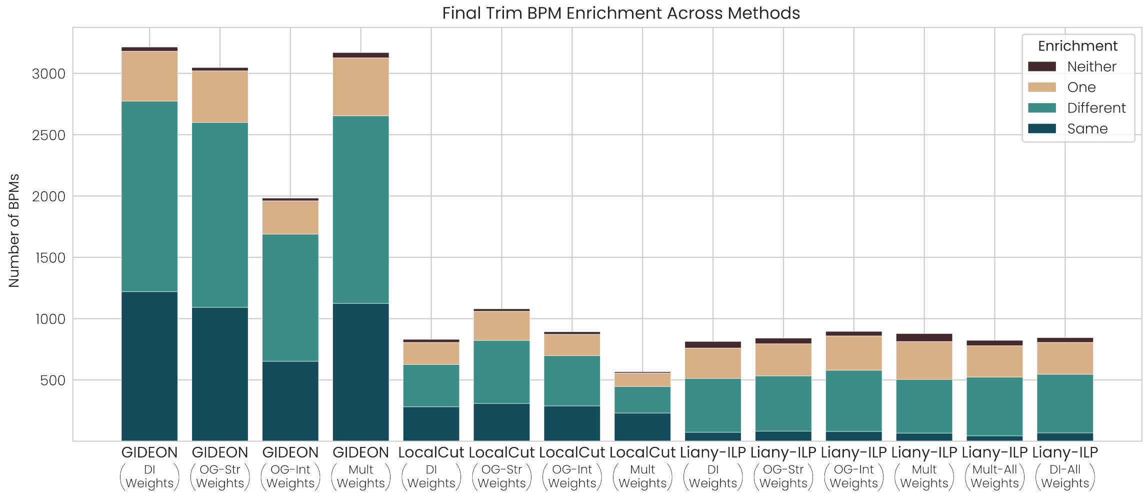

Figure S12: Finally, we compared GIDEON to new versions of LocalCut and Liany-ILP having these methods also apply the identical Final Trim step as GIDEON. Note that LocalCut loses BPMs through the Final Trim, demonstrating its prior performance is largely dependent on disconnected BPMs. Liany-ILP performance slightly improves, though BPM enrichment remains poor.

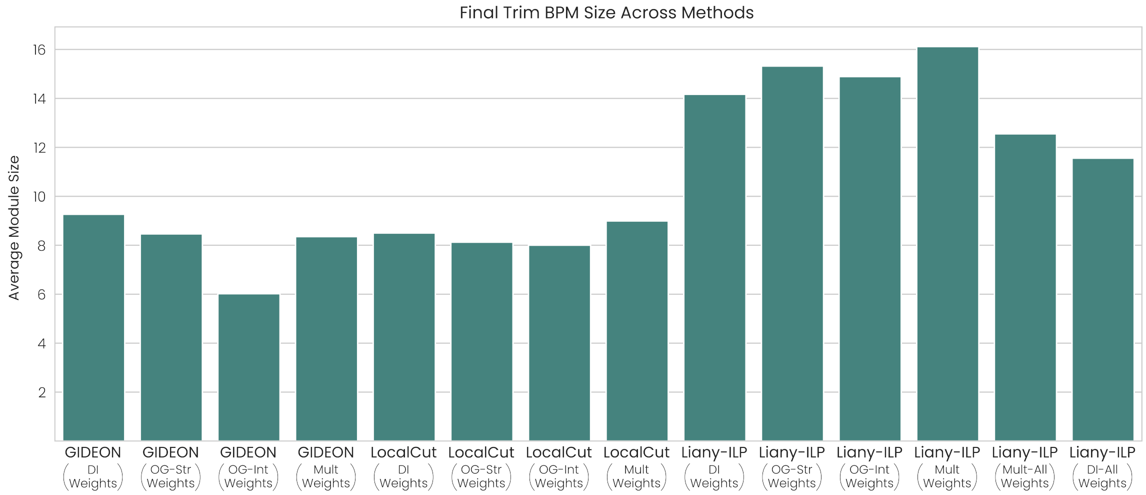

Figure S13: Compared with LocalCut and Liany-ILP after the Final Trim, GIDEON and LocalCut see an average of 8-9 genes per BPM than before while Liany-ILP begins to exhibit reasonable BPM sizes of 14-16 genes per BPM.

References

[1] M. Aebi, J. Gassenhuber, et al. Cloning and characterization of the ALG3 gene of *Saccharomyces cerevisiae*. *Glycobiology*, 6(4):439–444, 1996.

[2] L. Z. Ali and D. L. Sloan. Studies of the kinetic mechanism of hypoxanthine-guanine phosphoribosyltransferase from yeast. *Journal of Biological Chemistry*, 257(3):1149–1155, 1982.

[3] A. M. Bailis, L. Arthur, and R. Rothstein. Genome rearrangement in TOP3 mutants of *Saccharomyces cerevisiae* requires a functional RAD1 excision repair gene. *Molecular and cellular biology*, 12(11):4988–4993, 1992.

[4] F. Bianchi, J. S. van't Klooster, et al. Regulation of amino acid transport in *Saccharomyces cerevisiae*. *Microbiology and Molecular Biology Reviews*, 83(4):10–1128, 2019.

[5] J. M. Cherry, C. Adler, et al. SGD: Saccharomyces genome database. *Nucleic acids research*, 26(1):73–79, 1998.

[6] M. Costanzo, A. Baryshnikova, et al. The genetic landscape of a cell. *Science*, 327(5964):425–431, 2010.

[7] M. Costanzo, B. VanderSluis, et al. A global genetic interaction network maps a wiring diagram of cellular function. *Science*, 353(6306):aaf1420, 2016.

[8] J. S. Cox, C. E. Shamu, and P. Walter. Transcriptional induction of genes encoding endoplasmic reticulum resident proteins requires a transmembrane protein kinase. *Cell*, 73(6):1197–1206, 1993.

[9] K. Endo, Y.-i. Tago, et al. Error-free rad52 pathway and error-prone rev3 pathway determines spontaneous mutagenesis in saccharomyces cerevisiae. *Genes & genetic systems*, 82(1):35–42, 2007.

[10] D. G. Fisk, C. A. Ball, et al. *Saccharomyces cerevisiae* S288C genome annotation: a working hypothesis. *Yeast*, 23(12):857–865, 2006.

[11] L. C. Hancock, R. P. Behta, and J. M. Lopes. Genomic analysis of the Opi$^-$ phenotype. *Genetics*, 173(2):621–634, 2006.

[12] K. Jastrzebowska and I. Gabriel. Inhibitors of amino acids biosynthesis as antifungal agents. *Amino acids*, 47(2):227–249, 2015.

[13] T. Jordá and S. Puig. Regulation of ergosterol biosynthesis in *Saccharomyces cerevisiae*. *Genes*, 11(7):795, 2020.

[14] Y. Katou, Y. Kanoh, et al. S-phase checkpoint proteins Tof1 and Mrc1 form a stable replication-pausing complex. *Nature*, 424(6952):1078–1083, 2003.

[15] M. M. Kessler, Q. Zeng, et al. Systematic discovery of new genes in the *Saccharomyces cerevisiae* genome. *Genome Research*, 13(2):264–271, 2003.

[16] A. Kuplińska and K. Rzad. Molecular targets for antifungals in amino acid and protein biosynthetic pathways. *Amino Acids*, 53(7):961–991, 2021.

[17] M. D. Leiserson et al. Inferring mechanisms of compensation from E-MAP and SGA data using local search algorithms for max cut. *Jour. of Comp. Biol.*, 18(11):1399–1409, 2011.

[18] B. Llorente and B. Dujon. Transcriptional regulation of the *Saccharomyces cerevisiae* DAL5 gene family and identification of the high affinity nicotinic acid permease TNA1 (YGR260w). *FEBS letters*, 475(3):237–241, 2000.

[19] S. Mandel, K. Robzyk, and Y. Kassir. Ime1 gene encodes a transcription factor which is required to induce meiosis in *Saccharomyces cerevisiae*. *Developmental genetics*, 15(2):139–147, 1994.

[20] R. Mani, R. P. St. Onge, et al. Defining genetic interaction. *Proc. Natl. Acad. Sci. U.S.A.*, 105(9):3461–3466, 2008.

[21] J. R. Mullen, V. Kaliraman, et al. Requirement for three novel protein complexes in the absence of the Sgs1 DNA helicase in *Saccharomyces cerevisiae*. *Genetics*, 157(1):103–118, 2001.

[22] A. Paulovich, C. Armour, and L. H. Hartwell. The *Saccharomyces cerevisiae* RAD9, RAD17, RAD24 and MEC3 genes are required for tolerating irreparable, ultraviolet-induced DNA damage. *Genetics*, 150(1):75–93, 1998.

[23] P. Phi Poon, D. Cassel, et al. Retrograde transport from the yeast Golgi is mediated by two ARF GAP proteins with overlapping function. *The EMBO Journal*, 18(3):555–564, 1999.

[24] S. L. Squazzo, P. J. Costa, et al. The Paf1 complex physically and functionally associates with transcription elongation factors in vivo. *The EMBO journal*, 21(7):1764–1774, 2002.

[25] I. Stagljar, S. Te Heesen, and M. Aebi. New phenotype of mutations deficient in glucosylation of the lipid-linked oligosaccharide: cloning of the ALG8 locus. *Proceedings of the National Academy of Sciences*, 91(13):5977–5981, 1994.

[26] M. Sundström, Y. Lindqvist, et al. Yeast TKL1 gene encodes a transketolase that is required for efficient glycolysis and biosynthesis of aromatic amino acids. *Journal of Biological Chemistry*, 268(32):24346–24352, 1993.

[27] M. Usaj, Y. Tan, et al. TheCellMap. org: a web-accessible database for visualizing and mining the global yeast genetic interaction network. *G3: Genes, genomes, genetics*, 7(5):1539–1549, 2017.

[28] P. M. Watt, I. D. Hickson, et al. SGS1, a homologue of the Bloom's and Werner's syndrome genes, is required for maintenance of genome stability in *Saccharomyces cerevisiae*. *Genetics*, 144(3):935–945, 1996.

[29] J. R. Whyte and S. Munro. The Sec34/35 Golgi transport complex is related to the exocyst, defining a family of complexes involved in multiple steps of membrane traffic. *Developmental cell*, 1(4):527–537, 2001.

[30] K. M. Yu and L. J. Cowen. Exploring weighting schemes for the discovery of informative generalized between pathway models to uncover pathways in genetic interaction networks. *Scientific Reports*, 15(1):30169, 2025.
